# Analysis of the expression and distribution of protein O-linked mannose β1,2-*N*-acetylglucosaminyltransferase 1 in the normal adult mouse brain

**DOI:** 10.3389/fnana.2022.1043924

**Published:** 2023-01-06

**Authors:** Hanxiao Jiang, Yuxue Feng, Guiqiong He, Yuanjie Liu, Xiaofeng Li

**Affiliations:** ^1^Department of Neurology, The Second Affiliated Hospital of Chongqing Medical University, Chongqing, China; ^2^Chongqing Key Laboratory of Neurobiology, Chongqing Medical University, Chongqing, China; ^3^Department of Anatomy, Chongqing Medical University, Chongqing, China

**Keywords:** POMGNT1, O-mannosylation, neurons, brain distribution, adult mouse, Golgi

## Abstract

**Introduction:**

Protein O-linked mannose β1,2-*N*-acetylglucosaminyltransferase 1 (POMGNT1) is crucial for the elongation of O-mannosyl glycans. Mutations in POMGNT1 cause muscle-eye-brain (MEB) disease, one of the main features of which is anatomical aberrations in the brain. A growing number of studies have shown that defects in POMGNT1 affect neuronal migration and distribution, disrupt basement membranes, and misalign Cajal-Retzius cells. Several studies have examined the distribution and expression of POMGNT1 in the fetal or neonatal brain for neurodevelopmental studies in the mouse or human brain. However, little is known about the neuroanatomical distribution and expression of POMGNT1 in the normal adult mouse brain.

**Methods:**

We analyzed the expression of POMGNT1 mRNA and protein in the brains of various neuroanatomical regions and spinal cords by western blotting and RT-qPCR. We also detected the distribution profile of POMGnT1 in normal adult mouse brains by immunohistochemistry and double-immunofluorescence.

**Results:**

In the present study, we found that POMGNT1-positive cells were widely distributed in various regions of the brain, with high levels of expression in the cerebral cortex and hippocampus. In terms of cell type, POMGNT1 was predominantly expressed in neurons and was mainly enriched in glutamatergic neurons; to a lesser extent, it was expressed in glial cells. At the subcellular level, POMGNT1 was mainly co-localized with the Golgi apparatus, but expression in the endoplasmic reticulum and mitochondria could not be excluded.

**Discussion:**

The present study suggests that POMGNT1, although widely expressed in various brain regions, may has some regional and cellular specificity, and the outcomes of this study provide a new laboratory basis for revealing the possible involvement of POMGNT1 in normal physiological functions of the brain from a morphological perspective.

## Introduction

Human protein O-linked mannose β1,2-*N*-acetylglucosaminyltransferase 1 (POMGNT1) is a 660-amino-acid type II membrane protein (Larsen et al., 2017). The POMGNT1 gene, located at 1p34.1, is split into 22 exons, with the coding sequence beginning at exon 2 (Yoshida et al., 2001). POMGNT1 is a glycosyltransferase crucial for the elongation of O-mannosyl glycans in the brain, nerve, and skeletal muscle (Liu et al., 2017).

O-mannose glycans make up 30% of the known O-linked glycans in the brain (Hang et al., 2022). Various proteins need to be O-mannosylated before they can play their roles, such as α-myotrophiphylan (α-DG). Muscular dystrophy due to hypoglycosylation of α-DG is an autosomal recessive disorder in which muscle-eye-brain disease (MEB, or muscular dystrophy-muscular dystrophy glycosis type 3A) is primarily caused by homozygous or compound heterozygous mutations in the POMGNT1 gene (Mohammadi et al., 2021). It is clinically characterized by muscular dystrophy with eye and brain anomalies and intellectual disability. Substantial neuroradiologic structural brain abnormalities observed in dystroglycanopathy include pachygyria, polymicrogyria, lissencephaly, hydrocephalus, ventriculomegaly, agenesis of the corpus callosum, hypoplasia of the brainstem, hypoplasia, dysplasia, or cysts of the cerebellum (Fu et al., 2017; Mohammadi et al., 2021). In addition to MEB, POMGNT1 also participates in neuronal migration disorders (Gonzalez-Moron et al., 2017), the lissencephaly (Devisme et al., 2012), and the autism spectrum disorder (ASD) (Yu et al., 2013). In mouse models, deletion of POMGNT1 also leads to a variety of central nervous system abnormalities, including extensive changes in neuron distribution in the neocortex (Liu et al., 2010), neuronal overmigration during cerebral cortical development (Hu et al., 2007), a smaller cerebellum attributable to a reduced number of granule neurons and apparent cerebellar granule neuron ectopias (Li et al., 2008), as well as, a breached pial basement membrane and a defective dentate gyrus (Li et al., 2011). Hence, detailed brain distribution maps of POMGNT1 are the first step in revealing POMGNT1 in neuronal function which may help to characterize its role in the brain and ultimately understand the neuropathology of POMGNT1-associated diseases.

Preliminary research suggests *Pomgnt1* gene is found throughout the body such as the skeletal muscle, heart, brain, and kidney (Yoshida et al., 2001). However, little is understood about the regional distribution and cell type localization of POMGNT1 in the brain. Yamamoto et al. (2004) revealed that POMGnT1 cDNA was expressed in the spinal cord, cerebellum, and the cortical plate of the brain. And POMGNT1 was discovered in the neonatal rat brain microsome fraction (Takahashi et al., 2001). Despite that broad overview, an in-depth, detailed POMGNT1 protein brain localization is yet to be unraveled. Therefore, it is intriguing to observe POMGNT1 localized expression in the mouse brain which might provide a basis for the study of the neurophysiological function of POMGNT1.

In this study, we focused on characterizing the distribution profile of POMGnT1 in normal adult mouse brains by immunohistochemistry and double-immunofluorescence. We also analyzed the expression of POMGNT1 mRNA and protein in the brains of various neuroanatomical regions and spinal cords by western blotting and RT-qPCR. This study may promote the understanding of the physiological functions of POMGNT1 in the brain.

## Materials and methods

### Animals

Pathogen-free, 6-month-old, male C57BL/6J mice weighing (25–35 g) were maintained with sterile mouse chow and water *ad libitum* in the Animal Center of Chongqing Medical University with controlled temperature and light cycles (24°C and 12/12 light cycle). All experiments on mice were in accordance with was approved by the ethics committee of the Second Affiliated Hospital of Chongqing Medical University (NO.116/2021). The license number is SYXK(YU) 2018-0003. This experiment followed the RRR statement.

### Cell culture

Mouse hippocampal neuronal cells (HT22), mouse cerebellar astrocytes (MA-C) and mouse microglia (BV2) were cultivated in medium per the manufacturer’s instructions and maintained at 37°C in a humidified environment of 95% air and 5% CO_2_. Before conducting each experiment, cells were seeded in 35 mm plates and allowed to develop for 48 h at 37°C and 5% CO_2_.

### Brain tissue preparation

Mice (*n* = 10) are anesthetized using isoflurane, and after being sacrificed by decapitation, blood is rinsed with phosphate-buffered saline (PBS, pH 7.4). All procedures are performed on ice. The brain is split in two half, one half is immediately stored at −80°C for western blotting or RT-PCR, and the other half is post-immobilized in 4% paraformaldehyde (PFA). After dehydration of PFA-fixed brain tissue with fractionated ethanol, it is embedded in paraffin wax and then cut into 4 μm thick coronal or sagittal sections. Sections are mounted on glass slides for immunohistochemistry (IHC) and immunofluorescence (IF) staining.

### Immunohistochemistry staining

The paraffin embedded sections were deparaffinized, hydrated, and washed. Endogenous peroxidase activity was blocked in 3% H_2_O_2_ for 30 min without light. After washing, the sections were blocked with 3% BSA (Beyotime, Shanghai, China), and then immunostained with primary antibody (Table 1) overnight at 4°C. After being washed in PBS, the slices were incubated with biotinylated goat anti-rabbit IgG secondary antibody (Beyotime, Shanghai, China; 1:50 dilution) at room temperature for 50 mins followed by the application of a DAB Horseradish Peroxidase Color Development Kit (Beyotime, Shanghai, China). Harris hematoxylin counterstained for about 3 min, washed with tap water, differentiated with 1% hydrochloric acid alcohol for a few seconds, rinsed with tap water, blue with ammonia, rinsed with running water. After dehydration, the sections were photographed with Nikon Eclipse C1 microscope (Nikon). The hematoxylin stained nucleus is blue, and the positive expression of DAB is brownish-yellow. The immunohistochemical staining intensity of POMGNT1 protein was analyzed by semi-quantitative visual analysis using the standard 8-bit 16-color look-up table of ImageJ 1.51a (National Institutes of Health, Bethesda, MD, USA).

**TABLE 1:** Primary antibodies used in this work.

Protein	Antibody	Company	Working dilution		
			**WB**	**IHC**	**IF**
POMGNT1	Rabbit, pAb	Invitrogen, Carlsbad, CA, USA	1:1,000	1:100	1:200
β-actin	Rabbit, pAb	Proteintech, Wuhan, China	1:5,000	—	—
MAP2	Mouse, mAb	Signalway, Shanghai, China	—	—	1:200
S100B	Mouse, mAb	Santa Cruz, Dallas, TX, USA	—	—	1:200
GFAP	Mouse, mAb	Signalway, Shanghai, China	—	—	1:500
MBP	Mouse, mAb	CST, Massachusetts, USA	—	—	1:50
Iba-1	Mouse, mAb	GeneTex, Beijing, China	—	—	1:200
VGLUT1	Mouse, mAb	Abcam, Cambridge, UK	—	—	1:200
GAD65	Mouse, mAb	Abcam, Cambridge, UK	—	—	1:200
GM130	Mouse, mAb	Santa Cruz, Dallas, TX, USA	—	—	1:200
Calnexin	Mouse, mAb	Santa Cruz, Dallas, TX, USA	—	—	1:200
TOM20	Mouse, mAb	Abcam, Cambridge, UK	—	—	1:250

### Double IF staining

After deparaffinization in xylene and hydration by gradient alcohol, sliced, and mounted sections were incubated in Citrate Antigen Retrieval Solution (Beyotime, Shanghai, China) and then blocked for 30 min at room temperature in 8% normal goat serum. Sections were incubated overnight at 4°C with primary antibodies (Table 1). Alexa Fluor 488-labeled goat anti-rabbit IgG (H + L) (Beyotime, Shanghai, China; 1:400 dilution) and Cy3-labeled goat anti-mouse IgG (H + L) (Beyotime, Shanghai, China; 1:300 dilution) were used as secondary antibodies. The sections were incubated the secondary antibody at room temperature for 50 min in the dark and then covered with anti-fade mounting medium (Beyotime, Shanghai, China). After a thorough rinse with PBS, cover slips were mounted on the slides using 4′,6-diamidino-2-phenylindole (DAPI) (Beyotime, Shanghai, China) for cell nuclear counterstaining. Fluorescence images were captured using a Nikon Eclipse C1 microscope (Nikon). Colocalization was assessed with the JACoP plugin developed for ImageJ 1.51a (National Institutes of Health). Pearson’s correlation coefficient score (denoted by R) close to 1 was taken as indicative of complete colocalization and close to 0 of no colocalization.

### Western blotting

Mouse brain tissues and fresh cells were homogenized in RIPA lysis buffer (Beyotime, Shanghai, China) with PMSF (Beyotime, Shanghai, China). The supernatant was collected after centrifugation at 14,000 × g for 30 min at 4°C. The concentration of protein in the supernatant was quantified using an Enhanced BCA Protein Assay Kit (Beyotime, Shanghai, China) and all samples were diluted to equal concentrations using SDS-PAGE Sample Loading Buffer (Beyotime, Shanghai, China). Proteins (80 μg/lane) were separated by electrophoresis on 10% Tris-glycine polyacrylamide gels (Epizyme, Shanghai, China) and then transferred to polyvinylidene fluoride membranes. After blocking with 5% skim milk (Beyotime, Shanghai, China) with 0.1% Tween-20 for 2 h at room temperature, the membrane was incubated with primary antibodies (Table 1) in Primary Antibody Dilution Buffer (Beyotime, Shanghai, China) overnight at 4°C. After washing with TBST (TBS with 0.1% Tween 20) three times (5 min each time), the membrane was incubated with HRP-conjugated anti-rabbit IgG antibodies (Proteintech, Wuhan, China; 1:5,000 dilution), which was used as the secondary antibody, for 1 h at 37°C with gentle shaking. The membrane was washed three times in TBS with 0.01% Tween 20, each time for 20 min. Finally, the immunoreactive bands were enhanced by Hiper ECL Western HRP Substrate (Biosharp, Anhui, China) and then visualized using a Bio-Rad (Hercules, CA, USA) blot imager. Relative protein expression levels were calculated by Image J software (National Institutes of Health) normalized to ɑ-Tubulin.

### RNA extraction and RT-qPC

According to the manufacturer’s instructions, total RNA from mouse brain tissues was isolated using TRINzol Universal RNA Reagent (Tiangen, Beijing, China). The concentration was determined by the absorbance ratio at 260–280 nm using a NanoPhotometer^®^ spectrophotometer (Wilmington, DE, USA). cDNA synthesis was performed with PrimeScript™ RT Master Mix (TaKaRa, Otsu, Shiga, Japan) following the manufacturer’s T100™ Thermal Cycler (BIO-RAD, Hercules, CA, USA) instructions. RT-PCR was performed using a CFX Connect™ Real-Time System (BIO-RAD, Singapore) with forward and reverse primers. The *Pomgnt1* forward primer (5′–3′) was GCAAGACTATGATGAGGCCCTAG, and the reverse primer (5′–3′) was CATCCACAGCCACATACACTTTG. The *Gapdh* forward primer (5′–3′) was TGTTTCCTCGTCCCGTAGA, and the reverse primer (5′–3′) was ATCTCCACTTTGCCACTGC. According to the manufacturer’s instructions, the amplified cDNA was quantified using TB Green^®^ Premix Ex Taq™ II (Tli RNaseH Plus) (TaKaRa, Otsu, Shiga, Japan). Each reaction was performed with 8 μg template cDNA, 12.5 μl 2X TB Green Premix EX Taq II (Tli RNaseH Plus), 1 μl of each primer (10 μM), and water to adjust to a final volume of 25 μl. All reactions were incubated in a 96-well plate at 95°C for 30 s, followed by 40 cycles of 95°C for 5 s, 60°C for 30 s and melt curve to read the plate. Statistical analysis was performed using SDS software version 1.4.1 (Applied Biosystems). The housekeeping gene *Gapdh* was used to normalize the samples using the 2^–ΔΔ^*^C^*^T^ method.

### Statistical analysis

All experiments were performed at least in triplicates. The results were presented as the means ± SEM. Statistical comparisons were made using an analysis of variance followed by student’s *t*-test or one-way ANOVA. All data were analyzed using GraphPad Prism 8.0 (GraphPad Software, La Jolla, CA, USA).

## Results

### Expression of POMGNT1 in various regions of mouse brain

**FIGURE 1:**
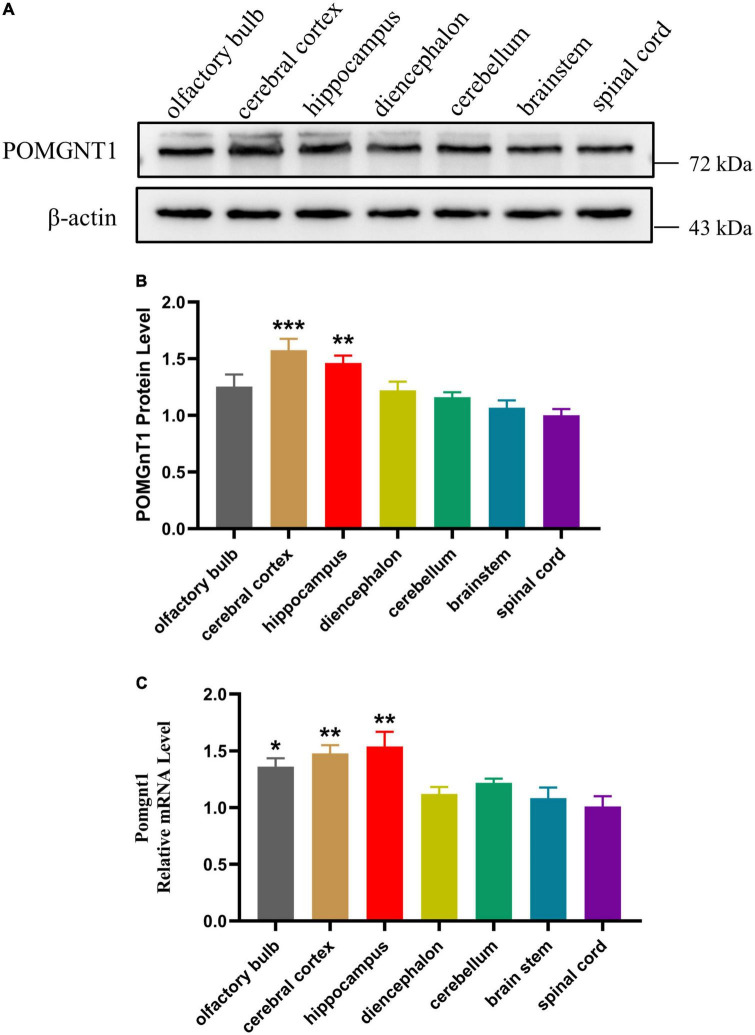
Expression of POMGNT1 in various regions of mouse brain. **(A)** Western blot was reprobed with mouse anti-β-actin antibody as the loading control. In total, 50 μg of protein was separated by SDS-PAGE transferred to a membrane, and probed with anti-POMGNT1 and anti-β-actin antibodies. **(B)** Quantifications showing the different protein expression levels of POMGNT1 in different brain regions. **(C)** RT-qPCR to measure the expression level of *Pomgnt1* in different regions of the brain and spinal cord. Data are presented as the mean ± SEM; *P < 0.05; **P < 0.01; ***P < 0.001 versus the spinal cord group (one-way ANOVA). *N* = 4.

To assess the expression of POMGNT1 in the various regions of adult mouse brain, we performed western blot and RT-qPCR analysis. We found the expression of POMGNT1 in cerebral cortex and hippocampus was significantly higher than that in spinal cord, with a mean 57.6 and 46.2% increase, respectively (Figures 1A, B). As shown in the Figure 1C, the expression level of *Pomgnt1* was similar in all regions of mouse brain except in olfactory bulb, cerebral cortex, and hippocampus, which showed a mean 35.3, 47, and 52.8% increase compared to spinal cord, respectively.

### Subregional distribution of POMGNT1 in the mouse brain

**FIGURE 2:**
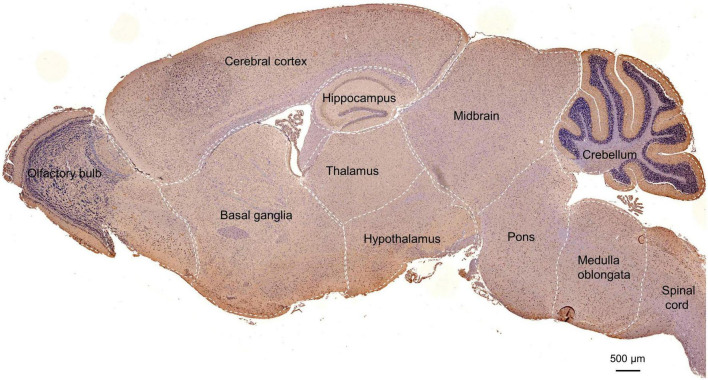
Overall POMGNT1 distribution patterns of the normal adult mouse brain. IHC staining of POMGNT1 in sagittal brain sections of the male mouse brain at 6 months. The hematoxylin stained nucleus is blue, and the positive expression of POMGNT1 is brownish-yellow. *N* = 6. Scale bar = 500 μm.

**FIGURE 3:**
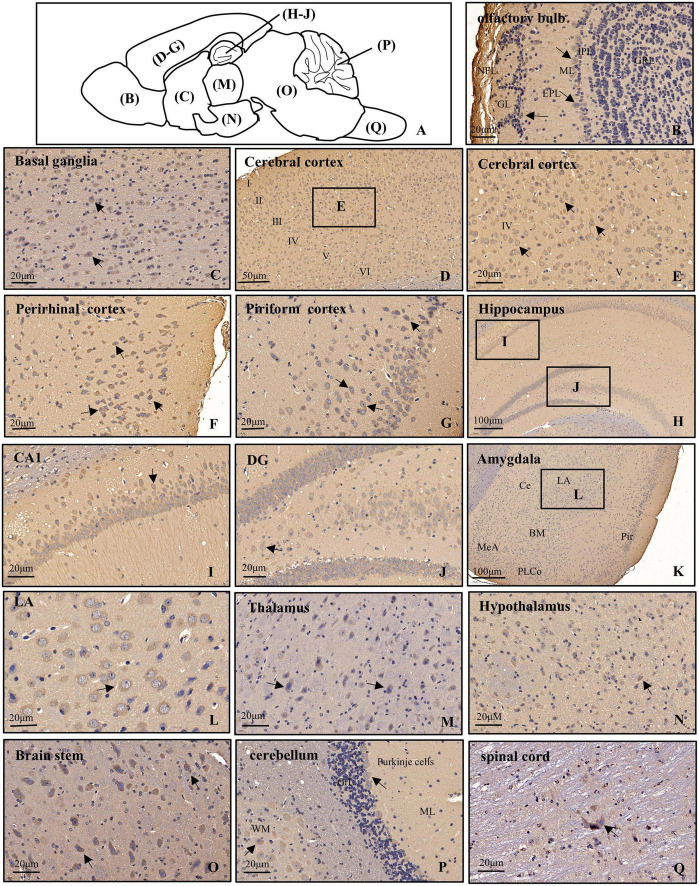
Distribution of POMGNT1 in the brain and spinal cord of the adult mouse. **(A)** Drawing with the location of the different brain regions shown in panels **(B–Q)**. The representative expression pattern of POMGNT1 in the coronal brain sections of the brain and spinal cord: **(B)** olfactory bulb, **(C)** basal ganglia, **(D–G)** cerebral cortex, **(H–J)** hippocampus, **(K,L)** amygdala, **(M)** thalamus, **(N)** hypothalamus, **(O)** brain stem, **(P)** cerebellum, and **(Q)** spinal cord. The hematoxylin stained nucleus is blue, and the positive expression of POMGNT1 is brownish-yellow. Arrows indicate representative POMGNT1-positive neurons. *N* = 6 mice. Scale bars = 20 μm **(B,C,E–G,I,J,L–Q)** or 50 μm **(D)**, or 100 μm **(H,K)**. NFL, olfactory nerve fiber layer; GL, glomerular layer; EPL, external plexiform layer; ML, mitral cell layer; IPL, internal plexiform layer; GRL, granule cell layer; DG, dentate gyrus; LA, lateral amygdaloid nucleus; CeA, central amygdaloid nucleus; BM, basomedial amygdaloid nucleus; MeA, medial amygdaloid nucleus; PLCo, posterolateral cortical amygdaloid nucleus; Pir, piriform cortex; ML, molecular layer; GrL, granular layer; WM, white matter.

To further examine POMGNT1 distribution patterns in the mouse brain, we performed IHC staining with POMGNT1 antibody on serial coronal or sagittal sections from mouse brain. Although the sagittal section’s staining revealed a widespread expression of POMGNT1 in the brain, the enrichment of POMGNT1-positive cells was observed in the cerebral cortex and hippocampus (Figure 2). Furthermore, we detected subregion-specific differences in POMGNT1 immunoreactivity (Figure 3A).

### Olfactory bulb

In the main olfactory bulb, moderate staining was mainly observed in the mitral cell layer (ML) and scattered in the glomerular layer (GL), while no POMGNT1-positive cell was observed in the olfactory nerve fiber layer (NFL), external plexiform layer (EPL), internal plexiform layer (IPL), or granule cell layer (GRL) (Figure 3B).

### Basal ganglia

In general, POMGNT1-positive cells were observed in various regions of the basal ganglia with strong to light staining intensity. We observed some strongly to moderately stained POMGNT1-positive cells located in the septal nucleus and the nucleus of the diagonal band. Extensive but light staining was observed in the striatum, including the caudate putamen and claustrum (Figure 3C).

### Cerebral cortex

Numerous POMGNT1-positive cells were distributed in various subregions of the cerebral cortex and stained from lightly to strongly. In general, the staining was heavy in the layers II–V and relatively light in the layers I and VI. We observed a host of pyramidal neurons located in the layers III and IV were moderately labeled. Some neurons with lightly labeled somatodendrites were seen in layer VI of the cortex. We also found a great number of polymorphic neurons were intensely labeled in the perirhinal cortex. In the piriform cortex, we observed intensely labeled POMGNT1-positive neurons were in the polymorphic layer along with a group of moderately labeled neurons (Figures 3D–G).

### Hippocampus

Abundant POMGNT1-positive neurons were observed in the hippocampus. The most prominent POMGNT1 labeling intensity was observed in the CA1-3 pyramidal cell layer of Ammon’s horn. All pyramidal cells with apical dendrites projecting into the adjoining stratum radiatum layer exhibited intensity staining. Some multipolar neurons labeled moderately were sporadically distributed in the strata oriens and strata radiatum. In the dentate gyrus, almost all granular cell somata were labeled moderately. We also observed moderately stained neurons in the hilus, while few POMGNT1-positive neurons in the adjacent molecular layer (Figures 3H–J).

### Amygdala

Some POMGNT1-positive cells with weak staining intensity were distributed in various subregions of the amygdala, including the central amygdaloid nucleus, lateral amygdaloid nucleus, basolateral amygdaloid nucleus, basomedial amygdaloid nucleus, and medial amygdaloid nucleus. However, cells located in the posteromedial cortical amygdaloid nucleus were mainly moderately labeled (Figures 3K–L).

### Thalamus and hypothalamus

POMGNT1-positive cells were distributed in most subregions of the thalamus with varying degrees of staining intensity. Strongly labeled cells were observed in the thalamic nucleus, including the central, posterior, and ventral group. Moderate to light staining was observed in the posterior paraventricular, mediodorsal, and the intermediodorsal thalamic nucleus. Negatively stained cells were also interspersed in the regions. We observed no immunoreactive cells in the habenular nucleus (Figure 3M). POMGNT1-positive cells were distributed in most subregions of the hypothalamus with weekly staining intensity, such as the hypothalamic nucleus, arcuate nucleus, perifornical nucleus, and medial tuberal nucleus. Some cells in the lateral hypothalamic area were also moderately stained (Figure 3N).

### Brainstem and cerebellum

In the brainstem, we also observed strong staining presented in some multipolar neurons with big nuclei, while the neurons with small nuclei nearly stained (Figure 3O). Overall, POMGNT1-positive neurons in the cerebellum were strongly labeled and located in the Purkinje cells and gray matter mass cells which spread among the white matter. We hardly found immunoreactive cells in the molecular layer or granular layer (Figure 3P).

### Spinal cord

Only a few POMGNT1-positive neurons with varying degrees of staining intensity were observed throughout the full length of spinal cord. The relatively strong stain was in the large neurons, whereas there was seldom staining in a subset of medium or small size neurons (Figure 3Q).

## Cellular localization of POMGNT1

**FIGURE 4:**
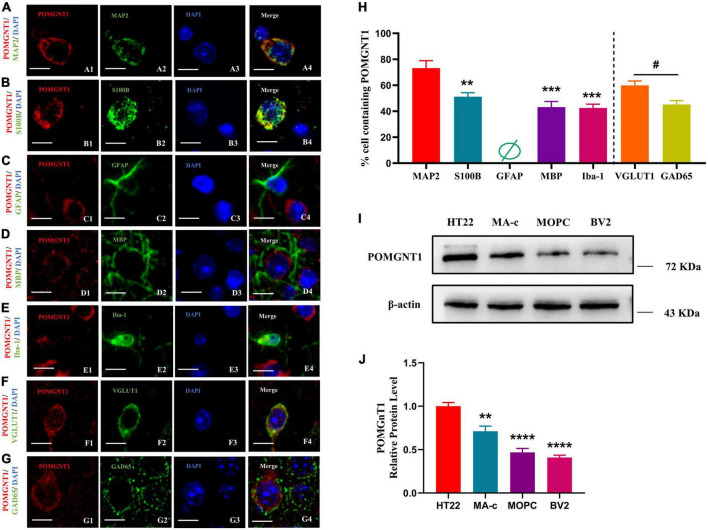
Cellular localization of POMGNT1. **(A–I)** Dual immunostaining of POMGNT1 (red) and neuronal glial cell subtype-specific markers (green) in the coronal brain sections of the cerebral cortex, including MAP2 for mature neurons **(A)**, S100B for resting and activated astrocytes **(B)**, GFAP for activated astrocytes **(C)**, MBP for oligodendrocytes **(D)**, or Iba-1 for microglia **(E)**, VGLUT1 for Glutamatergic neurons **(F)**, and GAD65 for GABAergic neurons **(G)**. POMGNT1 expressed in some neuronal glial cell subtype-specific marker-positive cells, including MAP2, S100B, MBP, Iba-1, GAD65, and VGLUT1, suggesting POMGNT1 may distribute in mature neurons, astrocytes, oligodendrocytes, microglia, glutamatergic neurons, and GABAergic neurons. Nearly no POMGNT1 expressed in the GFAP-positive cells suggesting POMGNT1 may not distribute in the activated astrocytes. *N* = 6. Scale bars = 20 μM. **(H)** Quantification of the percentage of the marker labeling cells containing POMGNT1 shown in panels **(A–G)**. *N* = 6. Data are presented as the mean ± SEM; **P < 0.01; ***P < 0.001 versus the MAP2 group (one-way ANOVA); ^#^*P* < 0.05 versus the VGLUT1 group (Student’s *t*-test). **(I)** The protein expression of POMGNT1 in neurons or glial cells using *in vitro* culturing, including HT22, MA-c, MOPC, and BV2, was detected by Western blot. β-actin as the loading control. **(J)** The results of the western blot were quantified. *N* = 4. Data are presented as the mean ± SEM; **P < 0.01; ****P < 0.0001 versus the HT22 group (one-way ANOVA).

The distribution pattern of POMGNT1 indicates that it might be expressed in neurons and neuroglia, as observed POMGNT1 staining in the pyramidal cells of the cerebral cortex (Figure 3E) and in the small, round cells of the basal ganglia (Figure 3C). To confirm whether POMGNT1 is expressed in the neurons or neuroglial cells, we performed double immunostaining using antibodies against POMGNT1 and either MAP2, S100B, GFAP, MBP, or Iba1 as specific markers for mature neurons, astrocytes, activated astrocytes, oligodendrocytes, or microglia, respectively. We found that the percentage of POMGNT1 immunostaining was highest in MAP2-positive mature neurons (73.17 ± 14.13%), higher in S100B-positive astrocytes (51.17 ± 7.65%), and lowest in MBP-positive oligodendrocytes (43.17 ± 10.67%), and Iba-1-positive microglia (42.25 ± 7.23%). Interestingly, the expression of POMGNT1 in GFAP-positive cells was so weak that we hardly found staining of POMGNT1 (Figures 4A–E, H). To further confirm the neurons and neuroglial cells localization of POMGNT1, we performed the western blot to detect the relative expression levels of POMGNT1 in neurons and glial cells using *in vitro* culturing. In accordance, neurons (HT22) expressed a larger amount of POMGNT1 than either astrocytes (MA-C), oligodendrocytes (MOPC) or microglia (BV2), as higher POMGNT1 expression level of neurons than either astrocytes, oligodendrocytes or microglia (28.83 ± 6.32, 53.13 ± 6.32, or 59.12 ± 6.32% lower than HT22, respectively) (Figures 4I, J).

To further evaluate which neuronal subpopulation POMGNT1 is expressed in, we carried out double immunostaining using antibodies against POMGNT1 and either VGLUT1 or GAD65 as specific markers for glutamatergic neurons or GABAergic neurons, respectively. We found the percentage of POMGNT1 immunostaining in VGLUT1-positive glutamatergic neurons (59.83 ± 8.57%) was significantly higher than that in GAD65-positive GABAergic neurons (45.17 ± 7.55%) (Figures 4F–H).

## Subcellular localization of POMGNT1

**FIGURE 5:**
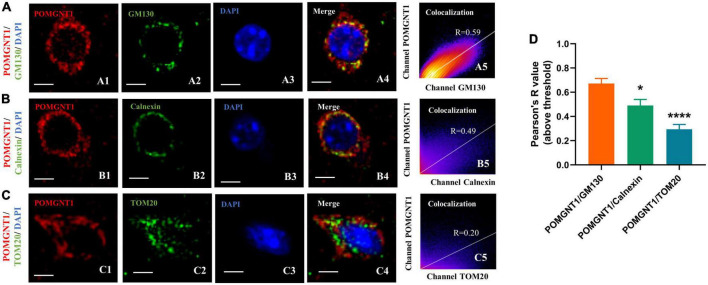
Subcellular localization of POMGNT1. **(A–C)** Dual immunostaining of POMGNT1 (red) and organelle-specific markers (green) in the coronal brain sections of the cerebral cortex. Merge images show their colocalization (yellow). Immunofluorescence imaging showing POMGNT1 co-localized with GM130 (Golgi marker) **(A)**, Calnexin (endoplasmic reticulum marker) **(B)**, and TOM20 (mitochondria marker) **(C)**. Co-localization is significantly highly in Golgi. Graphs in the right panel show Pearson’s correlation coefficient score (denoted by R) between POMGNT1 and organelle-specific marker for each image (0 < R < 1; A higher R-score indicates better co-localization between POMGNT1 and the organelle-specific marker). Scale bar = 20 μm. **(D)** Quantification of the Pearson’s correlation coefficient score between POMGNT1 and organelle-specific markers, GM130, Calnexin and TOM20, respectively. *N* = 6. Data are presented as the mean ± SEM; *P < 0.05; ****P < 0.0001 versus the POMGNT1/GM130 group (one-way ANOVA).

Our immunohistochemistry and fluorescence observations indicate that the POMGNT1 is mainly expressed in the soma of neurons. We also detected POMGNT1 immunostaining in neuronal processes, for instance, pyramidal dendrites in the cerebral cortex (Figure 3E). To better understand the intracellular POMGNT1 localization, we assessed its distribution inside soma of neurons by double immunostaining using GM130, Calnexin, and TOM20 to label the Golgi, endoplasmic reticulum (ER), and mitochondria, respectively. As shown in Figure 5, the majority of POMGNT1 was colocalized with GM130 (Rp = 0.67 ± 0.10, Figure 5A). We also found POMGNT1 and Calnexin were almost always colocalized (Rp = 0.49 ± 0.12, Figure 5B). In contrast, immunostaining with the mitochondrial marker TOM20 showed just a small overlap with POMGNT1 (Rp = 0.30 ± 0.09, Figure 5C).

## Discussion

Since structural phenotypes are probably linked to functional roles, it is crucial to characterize the detailed POMGNT1 expression profile in order to better understand how the protein works in the brain and to offer unique insights on pathologies linked to POMGNT1 (Kim et al., 2020). Although it has been reported that *Pomgnt1* mRNA is expressed in the cortex, cerebellum, and spinal cord of the human brain, a comprehensive and in-depth analysis of the distribution of POMGNT1 in the brain is still essential (Yamamoto et al., 2004).

In present study, we report for the first time the widespread distribution of POMGNT1 with significant regional specificity in the adult mouse brain, suggesting that this protein may play a variety of diverse functional roles in the brain. We detected extensive enrichment of moderate staining in the cortex and hippocampus, localized strong staining in the brainstem and cerebellum, and sporadic weak staining in the spinal cord. Since these specific brain neurons are engaged in memory formation and storage (Igarashi, 2022), autonomic and sensory activities (Elbaz et al., 2022), and motor coordination and execution (Fruzzetti et al., 2022), respectively. POMGNT1 may be predicted to have a substantial role in those events. Indeed, the unique brain distribution of POMGNT1 explains why various neuropsychiatric symptoms and CNS abnormalities are seen in MEB patients who have POMGNT1 mutations. For example, some patients present with significant intellectual disability, delayed language development, and trunk ataxia (Mohammadi et al., 2021), severe hypoplasia of the temporal lobe, corpus callosum, pons and cerebellum, dilated cerebral hemispheres and ventricular system, and abnormal bilateral frontal white matter signals (Fu et al., 2017; Borisovna et al., 2019; Mohammadi et al., 2021). As a result, the high to moderate levels of POMGNT1 expression in these brain regions also offer more proof of the protein’s significance in the nervous system as a whole.

Furthermore, it has been shown that the major components of the extracellular matrix of neurons in the mammalian brain are O-mannosylated and include calmodulin, the plexin superfamily, and perineuronal network proteins (Bartels et al., 2016). The perineuronal network (PNN) is a specialized structure of the extracellular matrix that surrounds the soma and proximal dendrites of certain neurons in the central nervous system and plays an important role in the study of plasticity and regeneration in the central nervous system (Magyar et al., 2022). Moreover, the unique spatial expression pattern of PNN in the brain is similar to that of POMGNT1 (Gaal et al., 2015; Kecskes et al., 2015; Hunyadi et al., 2020). Meanwhile, POMGNT1 is also involved in the functional role of extracellular matrix through α-DG (Wood et al., 2021). Then, whether POMGNT1 may perform corresponding functions through PNN, such as synaptic plasticity, is well worth exploring.

At the cellular level, we found that POMGNT1 was expressed in a variety of different neurons and glial cells, including mature neurons, astrocytes, oligodendrocytes and microglia, suggesting that POMGNT1 may play an important role in a variety of functional roles in the nervous system, such as, learning and cognition, myelin formation as well as neuroinflammation (Hitrec et al., 2021; Lujan et al., 2021; Ulloa-Navas et al., 2021). Consistent with this, it has been shown that POMGNT1 deficiency is associated with hearing impairment associated with impaired myelin formation and that POMGNT1 deficiency may lead to retinal reactive gliosis (Takahashi et al., 2011; Morioka et al., 2020).

Notably, POMGNT1 is mainly enriched in glutamatergic neurons. Glutamatergic neurons, the major excitatory neurons, secrete the excitatory neurotransmitter glutamate that controls synaptic plasticity in more than 40% of neurons in the brain (Conway, 2020). Therefore, the abundant expression of POMGNT1 in glutamatergic neurons helps to support this explanation for the synaptic impairments caused on by POMGNT1 deficiency (Nickolls and Bonnemann, 2018). In addition, studies have shown that glutamate has also been linked to the pathophysiology of Alzheimer’s disease (AD), which is characterized by the aggregation of Aβ and tau in the brain that damage learning and memory and prevent synaptic plasticity in neurons (Conway, 2020). Our previous study found POMGnT1 expression was decreased in AD models and that *in vitro* AD-like pathologies were improved by POMGNT1 overexpression (Feng et al., 2022). Therefore, it is critical to investigate into whether POMGNT1 affects synaptic plasticity in glutamatergic neurons and contributes to the pathogenesis of AD.

In our study, we hardly found POMGNT1 staining in GFAP-positive cells. However, Yamamoto et al. (2004) have found POMGnT1 was expressed in astrocytes by *in situ* hybridization. For *in situ* hybridization, the brains from two control fetal cases (22 and 23 gestational weeks) were also used. There are so many different in these two studies. The main difference is the brain sample, the former is the adult mouse brains, while the latter is the postmortem fetal cases. The subject age is also difference. The former is 6-month-old, while the latter is fetal (22 and 23 gestational weeks). Also, the former detected the protein POMGNT1 by the immunofluorescence, while the latter detected the cDNA POMGNT1 by *in situ* hybridization.

At the subcellular level, we discovered that POMGNT1 is distributed in the cytoplasm, similar finding was also observed in the mouse retina (Uribe et al., 2016). However, given that one study found POMGNT1 to be present in the nuclear fraction of the 661W photoreceptor cells, its presence in the nucleus cannot be fully ruled out (Uribe et al., 2016). As the nucleus is the most important cellular component in eukaryotic cells, it is necessary to further investigate the expression levels of POMGNT1 in nuclear proteins to verify if POMGNT1 is found in the nuclei of cells in mouse brain tissue. Furthermore, we found that POMGNT1 co-localizes with Golgi apparatus, ER and mitochondria to varying degrees, which may be related to its main functional role in cells. Several studies suggest that the main physiological function of POMGNT1 is involved in the O-mannosylation modification of α-DG by adding *N*-acetylamino glucose units to O-mannose in the Golgi apparatus (Tran et al., 2012; Bausewein et al., 2016). The main localization of POMGNT1 to the Golgi apparatus in the cortex of mouse brain is consistent with the putative function of this protein. Then, does POMGNT1 also function in the ER or mitochondria? Is this function known or unknown? More research is required.

**FIGURE 6:**
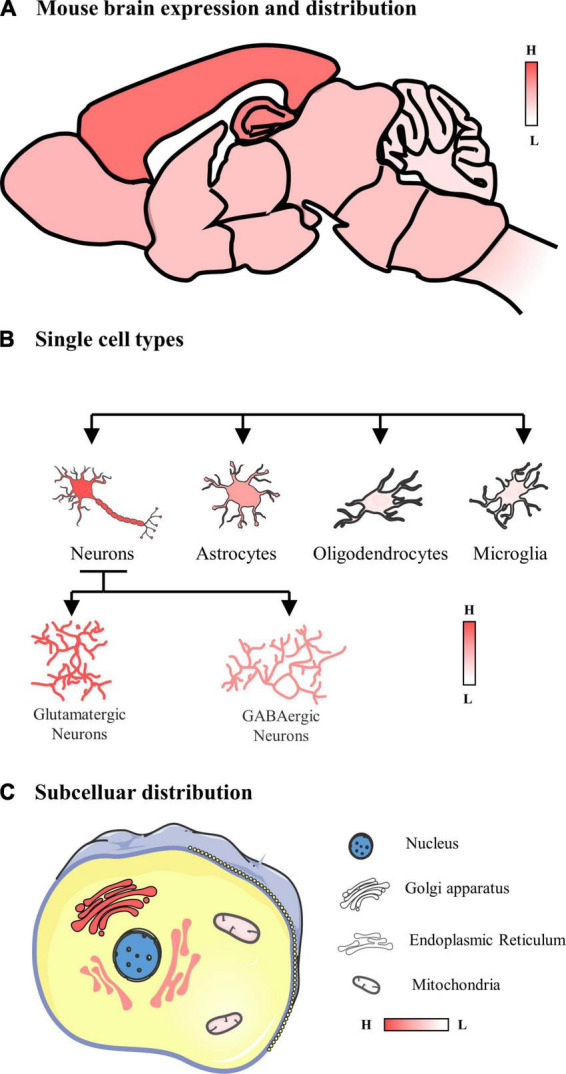
Schematic of the expression and distribution of POMGNT1 in the adult normal mouse brain. **(A)** The expression and distribution of POMGNT1 in the central nervous system of the adult mouse. POMGNT1 is widely distributed throughout the adult mice brain with regional specificity. Overall, POMGNT1 is enriched in the cerebral cortex and hippocampus. **(B)** The neuronal glial cell subtype specific distribution of POMGNT1. POMGNT1 was distributed in both neurons and neuroglia but they differed in the percentage of POMGNT1 positivity. The highest percentage of POMGNT1 positivity was found in mature neurons, higher in astrocytes, and least in oligodendrocytes and microglia. In contrast, the percentage of POMGN1 positivity was higher in glutamatergic neurons than in GABAergic neurons. **(C)** Subcellular specific localization of POMGNT1. POMGNT1 is expressed in the cytoplasm, mainly in the Golgi apparatus, followed by the endoplasmic reticulum, with the least in the mitochondria.

In summary, POMGNT1 is ubiquitously distributed in the normal adult mouse brain in a regional and cellular dependent manner (Figure 6). In order to identify POMGNT1’s role in the brain and ultimately comprehend the neuropathology of POMGNT1-associated illnesses like MEB, detailed brain distribution maps of POMGNT1 must first be created.

## Data availability statement

The original contributions presented in this study are included in the article/supplementary material, further inquiries can be directed to the corresponding authors.

## Ethics statement

All experiments on mice were approved by the Ethics Committee of the Second Affiliated Hospital of Chongqing Medical University (NO.116/2021). The license number is SYXK(YU) 2018-0003. This experiment followed the RRR statement.

## Author contributions

HJ designed the experiments with guidance from GH, YL, and XL, performed the experiments, and analyzed the data. HJ wrote the manuscript under the guidance of YF, GH, YL, and XL. All authors read and approved the final manuscript.
